# Beneficial Effects of Polydeoxyribonucleotide (PDRN) in an In Vitro Model of Fuchs Endothelial Corneal Dystrophy

**DOI:** 10.3390/ph15040447

**Published:** 2022-04-03

**Authors:** Ida Ceravolo, Federica Mannino, Natasha Irrera, Letteria Minutoli, Vincenzo Arcoraci, Domenica Altavilla, Gian Maria Cavallini, Salvatore Guarini, Francesco Squadrito, Giovanni Pallio

**Affiliations:** 1 Department of Clinical and Experimental Medicine, University of Messina, Via C. Valeria, 98125 Messina, Italy; ceravoloida@gmail.com (I.C.); fmannino@unime.it (F.M.); nirrera@unime.it (N.I.); lminutoli@unime.it (L.M.); varcoraci@unime.it (V.A.); gpallio@unime.it (G.P.); 2 Department of Biomedical, Dental, Morphological and Functional Imaging Sciences, University of Messina, Via C. Valeria, 98125 Messina, Italy; daltavilla@unime.it; 3 Institute of Ophthalmology, University of Modena and Reggio Emilia, Via del Pozzo, 41100 Modena, Italy; gianmaria.cavallini@unimore.it; 4 Department of Biomedical, Metabolic and Neural Sciences, Section of Pharmacology and Molecular Medicine, University of Modena and Reggio Emilia, Via G. Campi, 41125 Modena, Italy; salvatore.guarini@unimore.it

**Keywords:** Fuchs endothelial corneal dystrophy, ROS, oxidative stress, inflammation, apoptosis, polydeoxyribonucleotide

## Abstract

Fuchs endothelial corneal dystrophy (FECD) is a bilateral, hereditary syndrome characterized by progressive irreversible injury in the corneal endothelium; it is the most frequent cause for corneal transplantation worldwide. Oxidative stress induces the apoptosis of corneal endothelial cells (CECs), and has a crucial function in FECD pathogenesis. The stimulation of the adenosine A_2A_ receptor (A_2Ar_) inhibits oxidative stress, reduces inflammation and modulates apoptosis. Polydeoxyribonucleotide (PDRN) is a registered drug that acts through adenosine A_2Ar_. Thus, the goal of this study was to assess the effect of PDRN in an in vitro FECD model. Human Corneal Endothelial Cells (IHCE) were challenged with H_2_O_2_ (200 μM) alone or in combination with PDRN (100 μg/mL), PDRN plus ZM241385 (1 μM) as an A_2Ar_ antagonist, and CGS21680 (1 μM) as a well-known A_2Ar_ agonist. H_2_O_2_ reduced the cells’ viability and increased the expression of the pro-inflammatory markers NF-κB, IL-6, IL-1β, and TNF-α; by contrast, it decreased the expression of the anti-inflammatory IL-10. Moreover, the pro-apoptotic genes Bax, Caspase-3 and Caspase-8 were concurrently upregulated with a decrease of Bcl-2 expression. PDRN and CGS21680 reverted the negative effects of H_2_O_2_. Co-incubation with ZM241385 abolished the effects of PDRN, indicating that A_2Ar_ is involved in the mode of action of PDRN. These data suggest that PDRN defends IHCE cells against H_2_O_2_-induced damage, potentially as a result of its antioxidant, anti-inflammatory and antiapoptotic properties, suggesting that PDRN could be used as an FECD therapy.

## 1. Introduction

Fuchs endothelial corneal dystrophy (FECD) is a frequent corneal dystrophy with an unmet therapeutic approach. FECD has been documented in approximately 4% of individuals above the age of 40 years. It is characterized by gradual endothelial cell reduction, the thickening of the Descemet membrane, and localized excrescences known as guttae that normally begin in the middle of the cornea and gradually spread to the periphery [1]. Corneal endothelial cells (CECs) are involved in maintaining corneal transparency, deturgescence, and controlling corneal hydration. The loss of endothelial cells during FECD is irreversible because the corneal endothelium (CE) does not divide in vivo [2], thus leading to corneal oedema, scarring, and decreased visual acuity. The disease progresses in four stages, ranging from early symptoms of guttae development to end-stage subepithelial fibrous tissue deposition in response to chronic and protracted oedema. The typical symptoms are pain, photophobia and blurred vision, especially in the morning; the symptoms worsen, eventually leading to blindness, with the progression of the disease [3]. FECD is linked to a number of risk factors, including smoking, UV exposure, diabetes, family history, age and sex [4]. Previous studies have shown that oxidative stress plays a key role in the pathophysiology of FECD; in fact, increased levels of reactive oxygen species (ROS) were found in the corneas of FECD patients compared to healthy corneas [5,6]. Oxidative damage in the endothelium is caused by an imbalance between oxidant and antioxidant components that leads to ROS production, the dysregulation of pro- and antiapoptotic factors (Bax and Bcl-2), and the triggering of the apoptotic process mediated by Caspase 3 and 8, with consequent CEC loss in the corneas [7,8,9]. In addition, the increased expression of the transcription factor nuclear factor kappa-B (NF-κB), the subsequent release of the pro-inflammatory cytokine interleukin 6 (IL-6), and tumor necrosis factor alpha (TNF-α) activity have been linked to low CEC density and the development of symptomatic late-onset FECD [10]. TNF-α triggers programmed cell death and prompts the stimulation of the pro-inflammatory cytokine interleukin 1 beta (IL-1β), leading to chronic inflammation and the consequent injury of corneal endothelial tissue [11]. Despite the increased attention to the management of FECD, nowadays the nonsurgical therapy for FECD is palliative and limited to the topical application of hypertonic 5% sodium chloride eye drops. For this reason, finding new treatment approaches for the management of corneal diseases is of great interest. Adenosine receptors have been recognized as a promising target in the management of ROS-related disorders, eye diseases and impaired healing conditions [12,13,14,15,16]. In particular, adenosine A_2A_ receptor (A_2Ar_) activation showed the ability to modulate the inflammatory response and the apoptotic process, and to improve tissue repair and the healing process [17,18]. Polydeoxyribonucleotide (PDRN) is a biologic drug isolated from the gonads of trout, the antioxidant and anti-inflammatory effects of which have been demonstrated mainly through A_2Ar_ modulation [19,20]. Previous papers reported A_2Ar_ expression in the corneal endothelial cells [21], and that H_2_O_2_ stimulus in this cell line may cause significant alterations—such as apoptosis induction—similar to those observed in FECD [6]; for this reason, the goal of this work was to assess the effects of PDRN in an H_2_O_2_-induced in vitro FECD model.

## 2. Results

### 2.1. Effects of PDRN on Cell Viability

**Figure 1:**
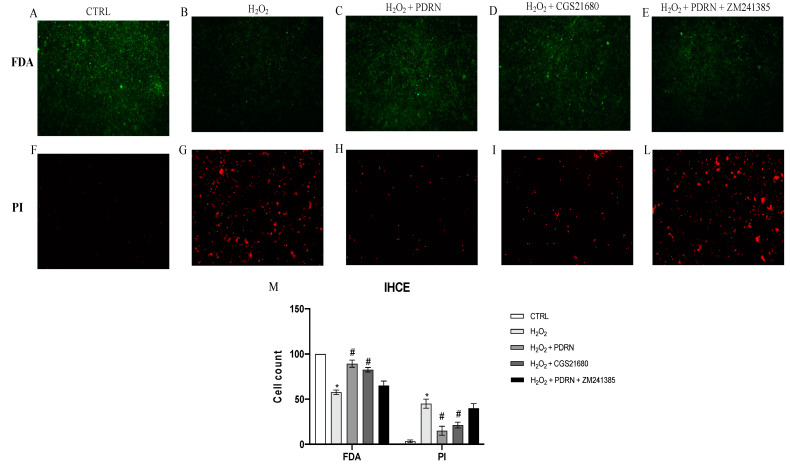
The figure shows the availability of the IHCE cell line treated with PDRN, as evaluated by FDA/PI staining. In panels (**A**–**E**) the green color staining indicates viable IHCE cells; in panels (**F**–**L**) the red staining indicates IHCE cells in apoptosis. Panel (**M**) shows the IHCE cell count. The data are expressed as means ± SD. * *p* < 0.0001 vs. CTRL; # *p* < 0.0001 vs. H_2_O_2_.

The control cells showed 100% viability, whereas cell exposure to H_2_O_2_ 200 μM for 2 h significantly reduced IHCE viability compared to the controls (*p* < 0.0001 vs. the control group; Figure 1). The IHCE cells’ viability following H_2_O_2_ challenge and treatment with PDRN for 24 h was extensively increased (*p* < 0.0001 vs. H_2_O_2_ group; Figure 1). Furthermore, the treatment with the specific A_2Ar_ agonist CGS21680 significantly augmented IHCE cells’ cell viability, thus confirming A_2Ar_’s involvement in the promotion of cell viability. The co-incubation with the A_2Ar_ antagonist ZM241385 abrogated the effects of PDRN, thus pointing out PDRN’s mechanism of action in this cell type and in an oxidative stress condition (*p* < 0.0001 vs. the PDRN group; Figure 1).

### 2.2. The Effects of PDRN on Oxidative Stress

**Figure 2:**
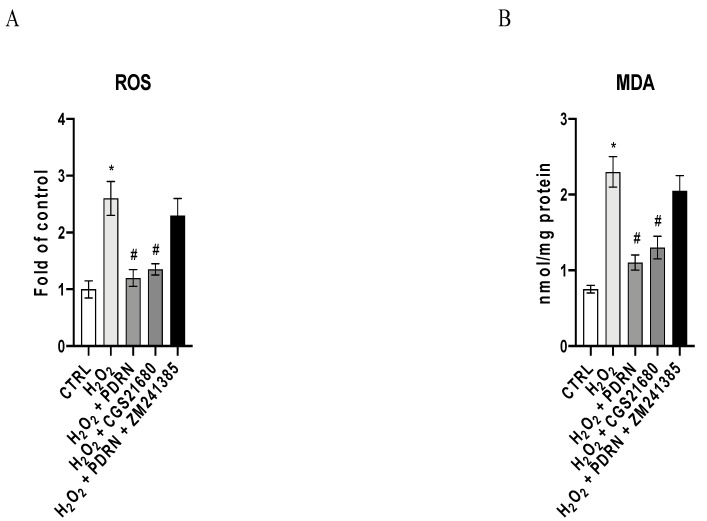
Effects of PDRN treatment on ROS production (**A**) and MDA generation (**B**) in IHCE cells. The values are expressed as the means ± SD. * *p* < 0.0001 vs. CTRL; # *p* < 0.0001 vs. H_2_O_2_.

A significant increase in ROS levels was observed in IHCE cells as a consequence of H_2_O_2_ stimulation (*p* < 0.0001 vs. the control group; Figure 2A). PDRN and CGS treatment significantly reduced ROS levels compared to untreated IHCE cells, confirming that A_2Ar_ stimulation is involved in the antioxidant effects of PDRN (*p* < 0.0001 vs. the H_2_O_2_ group; Figure 2A). The co-incubation with ZM241385 blunted the antioxidant effects of PDRN, confirming that PDRN acts through A_2Ar_ modulation (Figure 2A).

In order to better characterize the antioxidant effects of PDRN, the malondialdeyide (MDA) levels were measured in the IHCE cells. Low levels of MDA were detected in the control cells, whereas H_2_O_2_ challenge considerably increased the MDA levels (*p* < 0.0001 vs. the control group; Figure 2B). Both PDRN and CGS21680 decreased MDA production (*p* < 0.0001 vs. H_2_O_2_ group; Figure 2B), while co-incubation with ZM241385 counteracted the beneficial effects of PDRN (Figure 2B).

### 2.3. The Effects of PDRN on Inflammatory Markers

**Figure 3:**
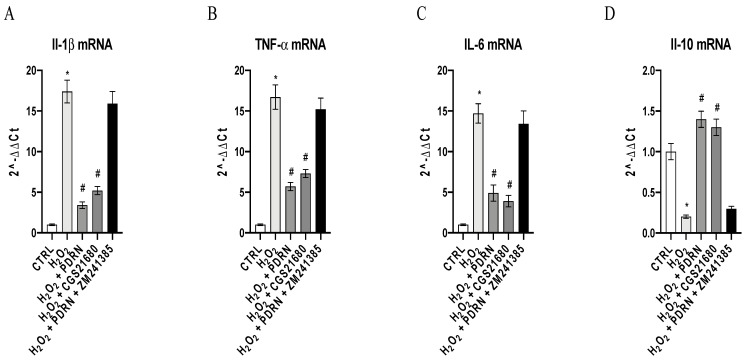
The graphs represent the qPCR results of Il-1β (**A**), TNF-α (**B**), Il-6 (**C**) and Il-10 (**D**) gene expression in IHCE cells. The values are expressed as the means and SD. * *p* < 0.0001 vs. CTRL; # *p* < 0.0001 vs. H_2_O_2_.

The gene expression of TNF-α, IL-1β, IL- 6 and IL-10 was studied in order to evaluate the anti-inflammatory effects of PDRN in this experimental model. H_2_O_2_ exposure caused a significant increase in TNF-α, IL-1β and IL- 6 gene expression, as well as a significant decrease of IL-10 expression compared to untreated cells (*p* < 0.0001 vs. the control group; Figure 3). PDRN treatment blunted TNF-α, IL-1β and IL-6 expression, and upregulated IL-10 mRNA expression compared to the H_2_O_2_ group (*p* < 0.0001 vs. the H_2_O_2_ group; Figure 3). CGS21680, a selective A_2Ar_ agonist, caused similar effects, indicating that A_2Ar_ is involved in inflammatory cascade modulation (*p* < 0.0001 vs. the H_2_O_2_ group; Figure 3). The co-incubation with the A_2Ar_ antagonist ZM241385 abrogated the effects of PDRN, thus corroborating the involvement of A_2Ar_ in PDRN’s mechanism of action (Figure 3).

**Figure 4:**
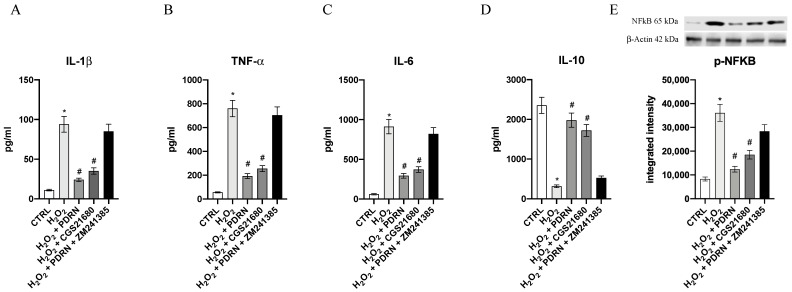
The graphs represent the Il-1β (**A**), TNF-α (**B**), Il-6 (**C**), Il-10 (**D**) and p-NF-κB (**E**) protein expression in IHCE cells. The values are expressed as the means and SD. * *p* < 0.0001 vs. CTRL; # *p* < 0.0001 vs. H_2_O_2_.

In addition, the expression of mature proteins was evaluated in order to confirm PDRN’s anti-inflammatory effects. TNF-α, IL-1β, p-NF-κB and IL-6 protein expression were markedly increased in H_2_O_2_-stimulated cells compared to the untreated cells (*p* < 0.0001 vs. the control group; Figure 4). PDRN treatment dampened TNF-α, IL-1β, p-NF-κB and IL-6 increased expression following H_2_O_2_ incubation (*p* < 0.0001 vs. the H_2_O_2_ group; Figure 4). Additionally, IL-10 protein expression was markedly reduced following H_2_O_2_ incubation compared to the untreated cells (*p* < 0.0001 vs. the control group; Figure 4). PDRN and CGS21680 treatments showed a significant increase of IL-10 protein levels, confirming that PDRN anti-inflammatory effects were related to A_2Ar_ activation (*p* < 0.0001 vs. the H_2_O_2_ group; Figure 4). The co-incubation with the A_2Ar_ antagonist ZM241385 reverted the effects of PDRN, indicating that A_2Ar_ activation is involved in PDRN’s mechanism of action (Figure 4).

### 2.4. Effects of PDRN on Apoptosis

**Figure 5:**
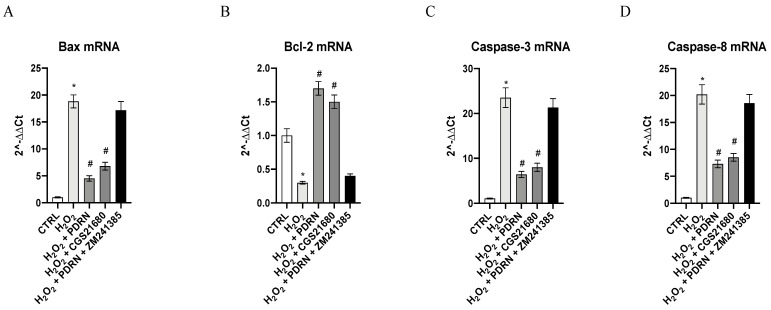
The graphs represent the gene expression results of Bax (**A**), Bcl-2 (**B**), Caspase-3 (**C**) and Caspase-8 (**D**) in IHCE cells. The values are expressed as the means and SD. * *p* < 0.0001 vs. CTRL; # *p* < 0.0001 vs. H_2_O_2_.

The gene expressions of Bcl-2, Bax, Caspase-3 and Caspase-8 were studied in order to evaluate the apoptosis process. IHCE cells challenged with H_2_O_2_ for 2 h exhibited a significant down-regulation of Bcl-2 mRNA levels with a simultaneous increase of Bax, Caspase-3 and Caspase-8 compared to the untreated cells (*p* < 0.0001 vs. the control group; Figure 5). On the other hand, PDRN treatment for 24 h completely inverted the gene expression of Bcl-2, Bax, Caspase-3 and Caspase-8 (*p* < 0.0001 vs. the H_2_O_2_ group; Figure 5). CGS21680, a selective A_2Ar_ agonist, showed similar effects, thus confirming the role of A_2Ar_ activation in the modulation of the apoptotic process (*p* < 0.0001 vs. H_2_O_2_ group; Figure 5). The A_2Ar_ antagonist ZM241385 counteracted the effects of PDRN, highlighting the role of A_2Ar_ in PDRN’s mode of action (Figure 5).

**Figure 6:**
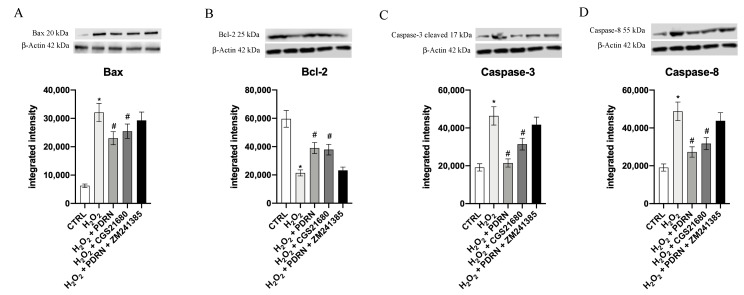
The graphs represent Bax (**A**), Bcl-2 (**B**), Caspase-3 (**C**) and Caspase-8 (**D**) protein expression in IHCE cells. The values are expressed as the means and SD. * *p* < 0.0001 vs. CTRL; # *p* < 0.0001 vs. H_2_O_2_.

In order to confirm the antiapoptotic effect of PDRN, we also evaluated the mature protein. A significant decrease of the Bcl-2 protein levels together with a concurrent increase of Bax, Caspase-3 and Caspase-8 were detected following H_2_O_2_ challenge compared to the untreated cells (*p* < 0.0001 vs. the control group; Figure 6). PDRN or CGS21680 treatment for 24 h caused a significant increase of Bcl-2 protein expression with a concomitant decrease of Bax, Caspase-3 and Caspase-8 protein levels (*p* < 0.0001 vs. the H_2_O_2_ group; Figure 6). ZM241385 blocked the effects of PDRN, again confirming the role of A_2Ar_ activation in apoptosis modulation (Figure 6).

## 3. Discussion

FECD is a bilateral heterogeneous disease characterized by the progressive loss of CECs, the production of localized excrescences known as guttae in the Descemet membrane, corneal oedema and impaired vision [3]. The loss of CECs observed in FECD is irreversible, as CE does not divide in vivo. Corneal transplantation is the only therapy option for the restoration of impaired vision, making FECD the most common cause of corneal-endothelial transplants worldwide [22]. Oxidative stress plays a key role in a variety of ocular disorders; in particular, chronic oxidative stress is likely to contribute to cellular and molecular damage in CECs, inducing apoptosis that causes CE degeneration, thus leading to FECD [6,23]. Previous papers have demonstrated that adenosine A_2Ar_ activation prevents ROS generation, also reducing inflammation and the apoptotic process [24,25]. PDRN, which is recognized as an A_2Ar_ agonist, showed several properties—including antioxidant, anti-apoptotic and anti-inflammatory ones—in several pre-clinical in vivo disease models, such as airway inflammation, cerebral ischemia, interstitial cystitis, acute lung injury, and ischemic colitis [26,27,28,29,30]. Moreover, a previous clinical trial demonstrated that PDRN eye drops stimulated corneal epithelium regeneration, thus supporting the hypothesis that it could be used for the treatment of corneal diseases [15,18]. Therefore, in the present study, the efficacy of PDRN was evaluated in an in vitro FECD model induced by H_2_O_2_ stimulation. Previous studies have demonstrated that hydrogen peroxide stimulation may promote the functional and structural changes that occur in corneal endothelial cells in FECD [6,31]. Furthermore, in this experimental setting, CECs challenged with H_2_O_2_ showed an increase of oxidative stress, inflammatory and apoptotic processes, in accordance with previous papers [32,33]. PDRN treatment drastically reduced oxidative stress markers such as ROS content and MDA levels compared to the H_2_O_2_ group. By contrast, these effects were abolished when the A_2Ar_ antagonist ZM241385 was used together with PDRN, thus demonstrating that its antioxidant effects occurred through adenosine A_2Ar_ stimulation. Moreover, CECs challenged with H_2_O_2_ and treated with the specific A_2Ar_ agonist CGS21680 showed a marked reduction of either ROS or MDA levels, thus confirming the involvement of A_2Ar_ in oxidative stress suppression in these experimental conditions. These results are consistent with previous articles that showed the protective effects of PDRN against ROS-related diseases, such as IBD [13], supporting the idea that the implementation of an antioxidant system through adenosine A_2Ar_ activation may also play a role in the treatment of diseases characterized by oxidative stress, such as FECD.

Moreover, H_2_O_2_ stimulation activated the transcriptional factor NF-κB, which in turn enhanced the expression of the pro-inflammatory cytokines TNF-α, IL-1β and IL-6, with a concomitant downregulation of the anti-inflammatory cytokine IL-10. PDRN significantly reduced pro-inflammatory cytokine expression and increased IL-10 levels compared to the untreated H_2_O_2_ group of cells. CECs treated with CGS21680 showed similar results, while ZM241385 co-incubation inhibited the beneficial effects of PDRN, demonstrating again that PDRN activity occurs through A_2Ar_ stimulation. These findings are consistent with earlier data, which have characterized well the anti-inflammatory effects mediated by A_2Ar_ in several preclinical inflammatory models [34,35], and with recent papers that pointed out the inactivation of the NF-κB pathway mediated by PDRN [36,37].

The activation of the oxidative stress mechanism may be also responsible for the induction of cell death processes; in fact, H_2_O_2_ stimulus induced apoptosis in CECs. PDRN treatment decreased the expression of the proteins involved in apoptosis—such as Bax, Caspase-3 and Caspase-8—whereas it increased Bcl-2 levels compared to untreated H_2_O_2_-stimulated cells. Furthermore, cells treated with CGS21680 showed a modulation of the apoptotic process; conversely, by adding ZM241385 to PDRN-treated cells, the positive effects observed when the compound was used alone were abolished, thus confirming that PDRN acts through modulation of adenosine A_2Ar_. As mentioned, these results are in line with the data obtained from previous studies, and indicate that adenosine A_2Ar_ modulation might prevent the worsening of oxidative stress-related diseases in eye models [38,39].

These preliminary findings are intriguing, and point to new therapeutic alternatives. However, a major limitation of the study is that the effects of PDRN were evaluated in an in vitro FECD model; hence, additional in vivo research would be required in order to characterize its efficacy. Instead, PDRN might be deemed to be a novel treatment for FECD, because this adenosine A_2Ar_ agonist is already on the market for a variety of uses and it could be readily available for a clinical trial in FECD patients. Moreover, previous works demonstrated that the half-life of PDRN is around 12–17 h, indicating that it may be suitable for once-daily administration in ordinary clinical practice [13]. Finally, it should be pointed out that PDRN is already commercially available, it is well tolerated, and it showed a very good safety profile across several clinical trials and in a variety of therapeutic applications [40,41,42]. These intriguing preclinical findings, in light of PDRN’s strong translational potential, should be confirmed in a FECD clinical context.

## 4. Materials and Methods

### 4.1. Cell Cultures

Human Corneal Endothelial Cells (IHCE) were purchased by Creative Biolabs neuroS London, UK. The IHCE cells were cultured in PriNeu I medium enriched with 10% Fetal Bovine Serum (FBS), recombinant human epidermal growth factor (5 ng/mL), a 5 μg/mL insulin plus 1% antibiotic mixture (penicillin/streptomycin), and G418 antibiotic in a humidified incubator at 37 °C and with a percentage of 5% CO_2_.

### 4.2. Cell Treatments

The IHCE cells were cultured in 6-well plates at a density of 3 × 10^6^ cells/well, and were challenged with 200 μM H_2_O_2_ (Sigma Aldrich, Milan, Italy) for 2 h to establish an oxidative stress model. After the H_2_O_2_ stimulus, the cells were treated with PDRN (100 μg/mL) (Placentex Integro, Mastelli Srl, Sanremo, Italy), CGS21680 (1μM A_2Ar_ agonist) (Tocris Bioscience, Bristol, UK) and PDRN (100 μg/mL) in combination with ZM241385 (1 μM A_2Ar_ antagonist) (Tocris Bioscience, Bristol, UK) for 24 h. The induction of the oxidative stress model, the doses and the experimental time were chosen according to previously published papers [6,14,16].

### 4.3. FDA/PI Staining

The cells were plated at a density of 5 × 10^5^ cells/well in a 24-well plate, and were incubated with H_2_O_2_ (200 μM) for 2 h; then, the cells were treated with PDRN (100 µg/mL), CGS21680 (1 μM) and PDRN + ZM241385 (1 μM) for 24 h. At the end of the treatment period, the cells were washed in sterile PBS and stained with the FDA/PI staining solution, with the addition of 3.2 μL FDA and 20 μL PI at the concentrations of 5mg/mL and 2 mg/mL, respectively, in 2 mL culture medium without FBS, for each well, for 5 min at room temperature in the dark. The viable cells were observed with a fluorescence microscope. ImageJ software for Windows was used to calculate the number of positive cells (Softonic, Barcelona, Spain).

### 4.4. ROS Measurement

In order to evaluate the effects of PDRN on oxidative stress, the production of total Reactive Oxygen Species (ROS) in the IHCE cells was measured using an assay kit (Thermo Fisher, Carlsbad, CA, USA), as previously described in detail [31].

### 4.5. Malondialdehyde Assay

The antioxidant effect of PDRN in IHCE cells was evaluated by measuring the malondialdehyde (MDA) levels, as previously described in detail [31].

### 4.6. Real-Time Quantitative PCR Amplification (RT-qPCR)

Il-1β, TNF-α, IL-6, IL-10, Bcl-2, Bax, Caspase-3 and Caspase-8 m-RNA expression was assessed as previously described. The primers used to identify both the targets and reference genes are listed in Table 1 [16].

### 4.7. Measurements of the Cytokines

The IL-1β, TNF-α, IL-6 and IL-10 levels were measured in the cell culture supernatants, using Enzyme-Linked Immunosorbent Assay (ELISA) kits (Abcam, Cambridge, UK), in agreement with the instructions given by the manufacturer [43,44,45].

### 4.8. Western Blot Analysis

After 24 h of treatment, the IHCE cells were collected and protein extraction was performed to evaluate pNF-κB, Bax, Bcl-2, Caspase-3 and Caspase-8 expression by Western Blot analysis, as previously described in detail [46].

### 4.9. Statistical Analysis

The data presented are the results of at least five experiments, and are expressed as the mean ± SD. In order to guarantee repeatability, all of the assays were carried out in duplicate. The differences between the groups were evaluated and analysed using one-way ANOVA with the Tukey post-test. A *p* value of less than 0.05 was considered significant. SPSS Statistics for Windows v22.0 was used for the statistical analysis (SPSS, Inc, Chicago, IL, USA), and GraphPad Prism was used to create the graphs (Version 8.0 for macOS, San Diego, CA, USA).

## 5. Conclusions

In conclusion, we demonstrated for the first time that PDRN through A_2Ar_ stimulation defends IHCE cells against H_2_O_2_-induced damage, potentially as a result of its antioxidant, anti-inflammatory and antiapoptotic properties, suggesting that PDRN could be used as an FECD therapy.

## Figures and Tables

**Table 1:** Primer list.

Gene	Sequence
β-actin	Fw:5′AGAGCTACGAGCTGCCTGAC3′
	Rw:5′AGCACTGTGTTGGCGTACAG3′
IL-1β	Fw:5′TGAGCTCGCCAGTGAAATGA3′
	Rw:5′AGATTCGTAGCTGGATGCCG3′
TNF-α	Fw:5′CAGAGGGCCTGTACCTCATC3′
	Rw:5′GGAAGACCCCTCCCAGATAG3′
IL-6	Fw:5′TTCGGTCCAGTTGCCTTCTC3′
	Rw:5′CAGCTCTGGCTTGTTCCTCA3′
IL-10	Fw:5′TGGCGCGGTGGATTCATAC3′
	Rw:5′AGGGGTCTGTTTTGTTGGCA3′
Bcl-2	Fw:5′GCTCTTGAGATCTCCGGTTG3′
	Rw:5′AATGCATAAGGCAACGATCC3′
Bax	Fw:5′TTTGCTTCAGGGTTTCATCC3′
	Rw:5′CAGTTGAAGTTGCCGTCAGA3′
Caspase-3	Fw:5′CCTGGTTCATCCAGTCGCTT
	Rw:5′ TCTGTTGCCACCTTTCGGTT
Caspase-8	Fw:5′GGTTAGGGGACTCGGAGACT3′
	Rw:5′CAGGCTCAGGAACTTGAGGG3′

## Data Availability

Data is contained within the article.
